# The *Clostridium difficile* Cell Wall Protein CwpV is Antigenically Variable between Strains, but Exhibits Conserved Aggregation-Promoting Function

**DOI:** 10.1371/journal.ppat.1002024

**Published:** 2011-04-21

**Authors:** Catherine B. Reynolds, Jenny E. Emerson, Lucia de la Riva, Robert P. Fagan, Neil F. Fairweather

**Affiliations:** Division of Cell and Molecular Biology, Centre for Molecular Microbiology and Infection, Imperial College London, London, United Kingdom; Dartmouth Medical School, United States of America

## Abstract

*Clostridium difficile* is the main cause of antibiotic-associated diarrhea, leading to significant morbidity and mortality and putting considerable economic pressure on healthcare systems. Current knowledge of the molecular basis of pathogenesis is limited primarily to the activities and regulation of two major toxins. In contrast, little is known of mechanisms used in colonization of the enteric system. *C. difficile* expresses a proteinaceous array on its cell surface known as the S-layer, consisting primarily of the major S-layer protein SlpA and a family of SlpA homologues, the cell wall protein (CWP) family. CwpV is the largest member of this family and is expressed in a phase variable manner. Here we show CwpV promotes *C. difficile* aggregation, mediated by the C-terminal repetitive domain. This domain varies markedly between strains; five distinct repeat types were identified and were shown to be antigenically distinct. Other aspects of CwpV are, however, conserved. All CwpV types are expressed in a phase variable manner. Using targeted gene knock-out, we show that a single site-specific recombinase RecV is required for CwpV phase variation. CwpV is post-translationally cleaved at a conserved site leading to formation of a complex of cleavage products. The highly conserved N-terminus anchors the CwpV complex to the cell surface. Therefore CwpV function, regulation and processing are highly conserved across *C. difficile* strains, whilst the functional domain exists in at least five antigenically distinct forms. This hints at a complex evolutionary history for CwpV.

## Author Summary

*Clostridium difficile* is a bacterial pathogen that causes antibiotic-associated diarrhea, which can be fatal. Infections often occur in healthcare facilities, where there is a high population density of susceptible patients with many possible routes of transmission. We currently do not know the functions of molecules found on the surface of *C. difficile*, which are likely to facilitate colonization of the host. In this study we characterize a protein called CwpV that is found on the surface of *C. difficile*. We show that bacteria isolated from different patients have different versions of CwpV and that the immune system's weapons (antibodies) against one version are useless against the others. This suggests that CwpV may help *C. difficile* to escape from our natural defenses. We have also found that CwpV promotes aggregation of *C. difficile*, which may be important for host colonization. To test these hypotheses in the future we will use animal models to compare genetically modified *C. difficile* expressing different levels of CwpV to see if they behave differently during infection. We hope that knowing more about CwpV will help guide improvements in the prevention and treatment of *C. difficile*.

## Introduction

*C. difficile* is gram-positive spore forming anaerobe and a major cause of antibiotic-associated diarrhea [1]. *C. difficile* infection (CDI) often occurs in the nosocomial environment where infection management exerts significant economic pressure on healthcare systems. The strong association of CDI with antibiotic usage reflects disruption of the normal gut flora allowing effective colonization by *C. difficile*. Strains causing disease produce one or two related toxins, TcdA and TcdB, which modulate the activity of host cell Rho GTPases, destroying the integrity of the epithelial cell barrier and inducing a variety of effects on intestinal cells [2]. Using isogenic mutants in a hamster model of infection, recent studies have shown that toxin B plays an important role in pathogenesis but the situation is less clear for TcdA [3], [4]. *C. difficile* strains causing CDI however are remarkably diverse. Despite a high prevalence of certain strain types, for example NAP1/027 in North America and Europe around 2005–6, no particular type dominates and strain prevalences vary geographically and temporally [5].

Factors expressed on the bacterial cell surface are likely to contribute to host colonization via interactions with host tissue, the immune system and other bacterial cells. In common with a large number of bacterial species [6], *C. difficile* produces an S-layer, a paracrystalline proteinaceous array that completely coats the bacterial cell wall. The *C. difficile* S-layer is primarily composed of two proteins, the high molecular weight S-layer protein (HMW SLP) and the low molecular weight (LMW) SLP. These proteins are derived from the precursor SlpA [7] through cleavage mediated by the cysteine protease Cwp84 [8], [9]. The LMW and HMW SLPs form a stable non-covalently associated complex, which has been analysed by small-angle X-ray scattering [10]. The HMW SLP contains putative cell wall binding motifs (Pfam 04122) which are thought to mediate attachment to the underlying cell wall. The LMW SLP is highly immunogenic and variable between strains, with over 20 distinct sequences identified to date [11], [12], [13], [14].

*C. difficile* 630 contains 28 paralogs of the HMW SLP [15]. Known as the cell wall protein (CWP) family, all members contain two or three copies of the putative cell wall binding motif in addition to a second unique domain that may specify function. Transcriptomic and proteomic studies have shown several of these proteins to be expressed *in vitro* [16], [17]. Antibodies against some of these proteins are found in serum of infected patients [18] implying at least some of the CWPs are expressed *in vivo* during infection and accessible to the host immune system.

In *C. difficile* 630 [15], CwpV is the largest member of the CWP family and consists of three distinct domains: (1) an N-terminal region with putative cell wall binding activity, (2) a region of unknown function terminating in a serine-glycine-rich flexible linker; (3) nine repeats of 120 amino acids each. We recently described the phase variable expression of CwpV and proposed a mechanism of control involving a *cwpV* switch [19]. Briefly, between the *cwpV* promoter and open reading frame there is a 195 bp invertible region flanked by imperfect 21 bp inverted repeats. In the OFF orientation this sequence adopts a stem loop terminator, preventing formation of full-length transcripts while in the other orientation (ON), the terminator is absent so full length *cwpV* transcription proceeds and CwpV is expressed.

In this study we investigate the regulation, post-translational processing and function of CwpV across a diverse panel of *C. difficile* strains. We definitively identify the site-specific recombinase necessary for CwpV phase variation, and show that the *cwpV* switch orientation is the primary determinant of CwpV expression. We show that the CwpV N-terminal domain is responsible for cell wall anchoring and is well conserved across strains. In contrast, we find high sequence variation of the C-terminal repeat sequences across *C. difficile* strains with five antigenically distinct repeat types identified. Despite this variability, both post-translational processing and function of CwpV appear to be conserved across *C. difficile* strains. All types of CwpV exhibit an aggregation-promoting function, which is conferred by the variable C-terminal domain. The implications of these findings relating to the role of CwpV in *C. difficile* infection are discussed.

## Results

### The recombinase RecV controls expression of CwpV in *C. difficile*

**Figure 1:**
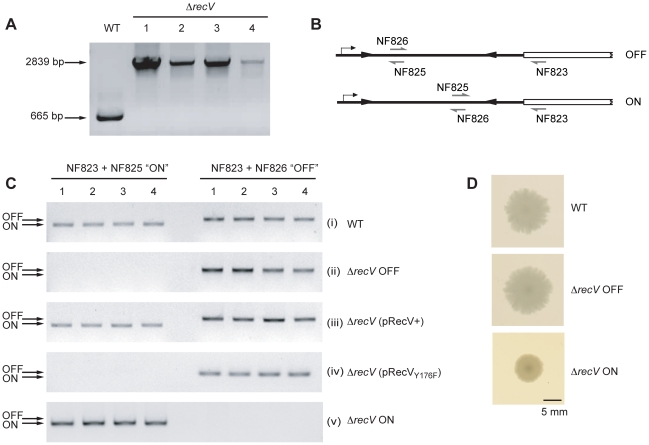
Isolation of Δ*recV* mutants. A. PCR confirmation of *recV* gene disruption using ClosTron. Primers NF1215+NF1356 flanking the ClosTron target site in *recV* give a 665 bp product in 630Δ*erm* (WT). After mutagenesis, four erythromycin resistant colonies were tested and a 2839 bp product was amplified, indicative of insertion of the group II intron into *recV*. These clones were designated Δ*recV* 1-4. **B.** Diagrammatic representation of the *cwpV* DNA switch orientation-specific PCR assay. Primers NF823+NF826 amplify a product from OFF, whilst primers NF823+NF825 amplify a product from ON. **C.** Analysis of the orientation of the *cwpV* DNA switch in *C. difficile* clones by orientation PCR. (i), products for the ON and OFF orientations are amplified from WT. (ii), all four isolated Δ*recV* mutants contain only the OFF orientation of the *cwpV* DNA switch, therefore these strains are referred to as 630Δ*recV* OFF. (iii), complementation of 630Δ*recV* OFF using a plasmid encoding *recV* (pRecV+) reconstituted the switching phenotype. (iv), a *recV*Y176F mutant was unable to complement Δ*recV* OFF confirming the key role of this tyrosine residue in RecV activity. (v), Δ*recV*(pRecV+) was serially sub-cultured without thiamphenicol selection to enable curing of the pRecV+ plasmid. Four thiamphenicol sensitive colonies were isolated from which only the ON orientation of the *cwpV* DNA switch could be amplified. These strains were therefore designated Δ*recV* ON. **D.** Colony morphologies of WT and Δ*recV* OFF are similar, however Δ*recV* ON exhibit a smaller, smoother-edged colony morphology.

Previously we identified the *C. difficile recV* gene as a strong candidate for the site-specific recombinase responsible for inversion of the *cwpV* DNA switch [19]. To definitively determine the role of *recV* in inversion of the *cwpV* switch, we constructed a *recV* knock-out in 630Δ*erm* using ClosTron technology [20]. 630Δ*erm* (wild-type) and four erythromycin resistant *recV* mutants, Δ*recV*1-4, were confirmed by PCR (Figure 1). To establish the orientation of the c*wpV* switch in these Δ*recV* strains, orientation-specific PCR was carried out [19] (Figure 1B). In wild-type clones, inversion of the *cwpV* switch was occurring as expected, shown by products with both primer pairs (Figure 1C i). All four Δ*recV* clones only amplified an OFF product (Figure 1C ii) and were therefore designated Δ*recV* OFF.

A plasmid encoding *recV* (pRecV+) was introduced into *C. difficile* Δ*recV* OFF by conjugation. The orientation of the *cwpV* switch in four Δ*recV*(pRecV+) transconjugants was tested by PCR. In all four strains, *cwpV* switch inversion was observed indicating successful complementation of the switching defect (Figure 1C iii). RecV is a member of the phage integrase family of tyrosine recombinases and by alignment tyrosine-176 was identified as a putative catalytic residue. To confirm the key role of this residue, we constructed a Tyr-Phe mutant (pRecV_Y176F_) which was introduced into Δ*recV* OFF. All four Δ*recV*(pRecV_Y176F_) transconjugants only had the *cwpV* switch in the OFF orientation (Figure 1C iv). Therefore RecV_Y176F_ could not complement the Δ*recV* OFF phenotype confirming the key role of tyrosine-176 in RecV- mediated inversion of the *cwpV* switch.

In order to isolate Δ*recV* ON mutants, Δ*recV*(pRecV+) was serially subcultured without thiamphenicol selection to allow loss of the pRecV+ plasmid. Thiamphenicol-sensitive colonies were tested for *cwpV* switch orientation and all were found to contain a single orientation, as the pRecV+ plasmid had been lost (data not shown). Four clones, from which only the ON orientation of the *cwpV* switch could be amplified, were isolated and designated Δ*recV* ON (Figure 1C v). The lack of inversion in these strains confirmed the necessary role of RecV in *cwpV* switch inversion, and suggests that this is the only recombinase responsible for inversion.

During the plasmid-curing process we noted two distinct colony morphologies amongst the resulting thiamphenicol-sensitive colonies. Interestingly, these were associated with the two orientations of the *cwpV* switch. In Figure 1D it can be seen that Δ*recV* OFF colonies have the same profuse, ruffled appearance as wild-type 630 colonies, whilst Δ*recV* ON colonies are smaller and smoother-edged. The hypothesis that CwpV expression in a Δ*recV* ON background causes a smaller colony phenotype was tested (see below).

### CwpV is the major CWP on *ΔrecV* ON cells

**Figure 2:**
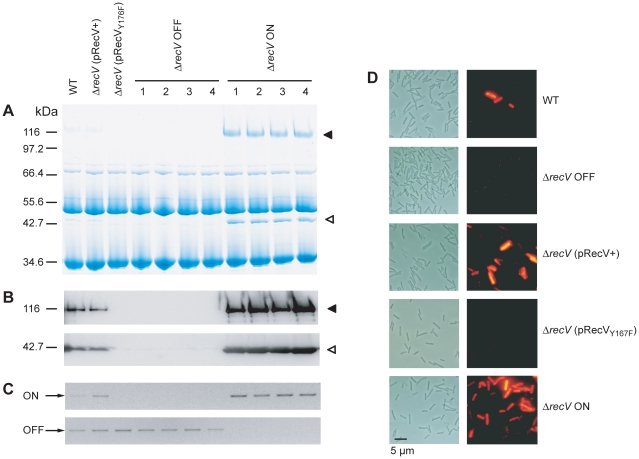
CwpV expression in Δ*recV* mutants. Overnight *C. difficile* cultures of WT, Δ*recV*(pRecV+), Δ*recV*(pRecV_Y176F_), Δ*recV* OFF (×4) and Δ*recV* ON (×4) were grown and analysed in the following ways: **A.** S-layer extracts were prepared and analysed by SDS-PAGE and Coomassie staining. **B.** Western blot of S-layer extracts using anti-CwpVrptI antibody (top; 1∶5000) and anti-CwpVNter antibody (bottom; 1∶5000). ◂, repeat domain; ◃, N-terminal domain. **C.** Orientation-specific PCR amplifying the ON and OFF orientations of the *cwpV* switch. **D.** Immunofluorescence analysis of expression of CwpV using anti-CwpVrptI antibody. Phase and fluorescence images of one field of view are shown.

The expression of CwpV in S-layer extracts of 630Δ*erm* (WT), Δ*recV*(pRecV*+*), Δ*recV*(pRecV_Y176F_), Δ*recV* OFF and Δ*recV* ON was analyzed by SDS-PAGE (Figure 2A). In WT and Δ*recV*(pRecV*+*) extracts, the two cleavage products of CwpV, the ∼42 kDa (N-terminal fragment) and ∼116 kDa (repeat domain) can be seen. However, as CwpV is only expressed by the minority of ON cells, the overall level of expression in these cultures is low. No expression of CwpV is seen in Δ*recV*(pRecV_Y176F_) or Δ*recV* OFF strains. Compared to WT cultures, Δ*recV* ON strains express a high amount of CwpV. Densitometry analysis indicated that in Δ*recV* ON strains, CwpV constituted 13.3% of the protein present in the S-layer (data not shown). These findings were confirmed by Western blot using anti-CwpVrptI (detects the ∼116 kDa CwpV repeat domain) and anti-CwpVNter (detects the ∼42 kDa N-terminal fragment) antibodies (Figures 2C). In all cases, expression of CwpV corresponded to *cwpV* switch orientation (Figure 2C).

In order to assess CwpV expression at the individual cell level, *C. difficile* cultures were stained using CwpVrptI antibody and analyzed by fluorescence microscopy (Figure 2D). In the WT culture, ∼5% of cells are ON as previously reported [19]. In Δ*recV* OFF, expression cannot be detected on any cell. In Δ*recV*(pRecV+) cultures, phase variable expression of CwpV can be seen with approximately 15% of cells expressing CwpV. The higher proportion of CwpV-positive cells is probably due to the altered level of RecV expression from the multi-copy plasmid, pRecV+. In Δ*recV* OFF(pRecV_Y176F_) no CwpV expression can be detected, as expected due to a lack of switch inversion from OFF. In Δ*recV* ON, CwpV expression can be detected in all cells. This suggests that *cwpV* switch orientation is the primary determinant of CwpV expression.

### The CwpV C-terminal repeat region is variable across *C. difficile* strains

**Figure 3:**
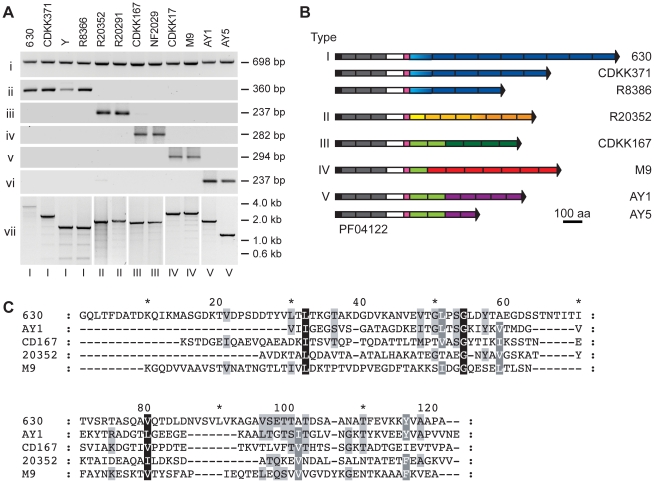
The CwpV C-terminal repeat region is variable across *C. difficile* strains. **A.** PCR analysis of the *cwpV* locus from diverse strains of *C. difficile*. Primers used were specific for: **i**, part of the conserved N-terminal domain (NF654 and NF655) **ii**, a single type I repeat (NF374 and NF346) **iii**, a single type II repeat (NF701 and NF702) **iv**, a single type III repeat (NF796 and NF797) **v**, a single type IV repeat (NF799 and NF800) **vi**, a single type V repeat (NF878 and NF879) **vii**, the entire repeat region, specifically amplified for each type (NF795 with NF373, NF703, NF798, NF801 or NF880). The *cwpV* type is indicated below each lane and the strain designation above. Details of the primers used are in Table S1. **B.** Cartoon representation of the eight different discovered configurations of CwpV proteins. For each combination of CwpV type and number of repeats, the repeat region from at least one strain was entirely sequenced. Black, signal sequence; grey, PF04122 cell wall anchoring domains; white, unknown function; pink, serine/glycine-rich region; blue, type I repeats; orange, type II repeats; green, type III repeats; red, type IV repeats, purple, type V repeats. Colour shade represents different sequence variants of a repeat type. Sequence in all strains is almost identical from the signal peptide through to the serine/glycine-rich region. Sequences were deposited at EMBL with accession numbers FM17250-8. **C.** CLUSTAL multiple sequence alignment of one repeat of each type from strains 630 (type I), 20352 (type II), CD167 (type III), M9 (type IV) and AY1 (type V).

Given the clinical importance of *C. difficile* strain diversity we sought to determine the level of conservation of *cwpV* across a variety of strains. PCR analysis of a collection of *C. difficile* strains using primers specific for the 5′ region of *cwpV* revealed the presence of a common fragment in all strains (Figure 3Ai). However, amplification using primers specific for the 630 repeat region generated products only in a subset of strains (Figure 3A ii). We have previously shown that, in these strains, the *cwpV* gene is highly similar to that of 630, but differs in the number of C-terminal repeats [19] (Figure 1A, panel vii). CDKK371 contains six repeats and strains R8366 and Y each contain four repeats. The repeats, termed type I, are highly similar to each other in DNA and predicted amino acid sequence, both within any one strain and between strains. In each strain the first repeat is more divergent from the remaining repeated sequences, although it is still almost identical between strains.

We were unable to amplify the 3′ region of *cwpV* from the remaining strains studied using primers designed against the 630 type I repeats (Figure 3A ii), consistent with a comparative genomic microarray study using DNA probes from strain 630 which showed that, while the 5′ region of *cwpV* is present in each of 75 strains studied, the 3′ region was only detected in 17 strains [21]. PCR amplification of the 3′ end of the *cwpV* gene from two ribotype 027 strains, R20352 and R20291, was achieved using primers found to be conserved in the 5′ end of the R20352 *cwpV* gene and in CD0515, the gene located downstream of *cwpV* in 630. The amino acid sequence of R20352 CwpV was almost identical to 630 CwpV from the N-terminus to the serine/glycine-rich region. However, instead of containing repeats of 120 amino acids, these 027 strains contained eight 79 amino acid repeat regions. PCR analysis with primers specific for the R20352 CwpV repeats is shown (Figure 3A iii and vii). These repeats, designated type II, were completely distinct from type I repeats. Several 027 strains were analyzed and the repeats were virtually identical in sequence to one another with the exception of the first repeat, which was slightly divergent from the consensus but conserved between strains.

In other *C. difficile* strains, where the 5′ region of the gene was present but the 3′ region could not be amplified, genomic DNA was digested with AseI and then re-ligated to form circular fragments. Following inverse PCR, sequence analysis revealed *cwpV* from all strains to be practically identical to 630 *cwpV* extending to the serine/glycine-rich region. The 3′ region of *cwpV* from strains CDKK167 and NF2029 encode six repeats of 94 amino acids distinct in sequence from types I and II, and were designated type III. These repeats could be amplified using repeat-specific primers (Figure 3A iv and vii). Two toxin A-B+ strains, CDKK17 and M9, were found to have a fourth type of repeat sequence. The first repeat is very similar to the first type III repeat, whereas the remaining seven repeats of 98–100 amino acids are distinct from all other repeats and were designated type IV. Finally, non-toxic strains, AY1 and AY5, were found to have a combination of type III repeats and a further novel 79 amino acid repeat, designated type V. Primers designed against type IV and V repeats reflected the sequence analysis, shown in Figure 3A v–vii.

It should be emphasized that the five types of CwpV repeat are completely unrelated to one another. A diagrammatic representation of the eight unique CwpV protein architectures discovered so far is shown in Figure 3B. A multiple sequence alignment of the amino acid sequences of one repeat of each type is shown in Figure 3C, illustrating visually how unrelated the sequences are as no meaningful alignment can be made.

Having characterized these five repeat types, a larger collection of strains were analyzed by PCR to determine their repeat types (see Table 1). Our analysis of strains was not exhaustive, and it seems possible that further CwpV repeat types may be present amongst the considerable diversity of the *C. difficile* species. However, we focused on the characterization of these five known CwpV repeat types. For each type, a model strain was decided upon and used to represent that type for the rest of the study. We chose; for type I 630, for type II R20352, for type III CDKK167, for type IV M9 (which also contains one type III repeat) and for type V AY1 (which also contains two type III repeats). A full multiple sequence alignment of all the repeat sequences is shown in Figure S1.

**Table 1:** CwpV characteristics of *C. difficile* strains.

Strain	CwpV architecture		Other characteristics		Reference or source
	Type	No. of repeats^a^^,^ b	Ribotype	Toxin status	
CDKK371	I	6^b^	001	+ve	[11]
R8366	I	4^b^	001	A+, B+	[7]
R13537	I	6^a^	001	+ve	[14]
R12879	I	6^a^	001	+ve	[14]
R14628	I	6^a^	001	+ve	[14]
Y	I	4^b^	010	A−, B−	[11]
630	I	9^b^^,^ c	012	A+, B+	[7]
CDKK959	I	4^b^	053	+ve	[11]
CDKK101	I	4^b^	053	+ve	[11]
CDKK291	I	4^b^	053	+ve	[11]
M120	I	4^a^	078	A+, B+	B. Wren
C22	I	4^a^	078	A+, B+	S. d'Arc
C36	I	4^a^	078	A+, B+	S. d'Arc
H57	I	4^a^	078	A+, B+	S. d'Arc
H94	I	4^a^	078	A+, B+	S. d'Arc
R20291	II	8^b^^,^ c	027	A+, B+; CDT+	[21]
R20352	II	8^b^	027	A+, B+; CDT+	[21]
R20928	II	8^a^	027	A+, B+; CDT+	[21]
CD196	II	8^a^	027	A+, B+; CDT+	[41]
R12628	II	8^a^	027	+ve	J. Brazier
R16760	II	8^a^	027	+ve	J. Brazier
CDKK167	III	6^b^	016	+ve	C. Kelly
NF2029	III	6^a^	106	A+, B+	M. Wilcox
R7404	III/IV	1/7	017	A−, B+	[7]
M9	III/IV	1/7^b^	017	A−, B+	D. Drudy
AY1	III/V	2/5^b^	N.D.	A−, B−	D. Gerding
AY2	III/V	2/5^b^	N.D.	A−, B−	D. Gerding
AY3	III/V	2/5^b^	N.D.	A−, B−	D. Gerding
AY4	III/V	2/5^b^	N.D.	A−, B−	D. Gerding
AY5	III/V	2/2^b^	N.D.	A−, B−	D. Gerding
B-one (B1)	I	9^a^	N.D.	A+, B+	Gill Douce; [42]

The types of repeats and their number were determined by

a PCR (this study),

b DNA sequencing (this study) or

c genome sequencing studies http://www.sanger.ac.uk/resources/downloads/bacteria/clostridium-difficile.html; N.D., not determined; +ve, toxin A+ve but toxin B unknown.

### Different CwpV repeat types are antigenically distinct

**Figure 4:**
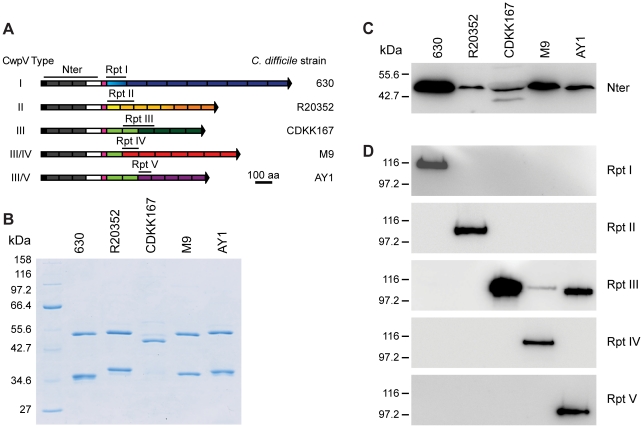
CwpV repeat types are antigenically distinct. **A.** Cross-reactivity of *C. difficile* S-layer extracts from strains of different CwpV type. Recombinant CwpV domains were expressed and purified from *E. coli* for use as antigens. The domains used are illustrated by black lines above the cartoons of CwpV proteins. **B.** S-layer extracts from *C. difficile* strains analysed by SDS-PAGE illustrates the variability of S-layer proteins across strains, as previously described [11]. **C.** Western blot of S-layer extracts using anti-CwpVNter shows that this domain is recognized in all strains, as expected due to high sequence conservation. **D.** Western blots of S-layer extracts using antibodies against CwpV repeat types I–V show that each repeat-specific antibody only detects CwpV proteins containing at least one repeat of its own repeat type.

Given the completely different amino acid sequences for each repeat type we hypothesized that the repeats may be antigenically distinct. CwpV expression was therefore investigated using antibodies raised against recombinant CwpV domains. The CwpV domains used as antigens are indicated by black lines above the protein cartoons in Figure 4A. S-layer extracts were prepared for the representative strain of each type and were visualized by SDS-PAGE (Figure 4B). The significant variation in SlpA across these strains can be seen, as described previously [11]. Analysis by Western blotting with anti-CwpVNter antibody reveals a single band at ∼42 kDa in each strain (Figure 4C) as would be expected by the high level of conservation of the CwpV N-terminal domain across strains. This confirmed expression of CwpV and localization to the cell wall in all these strains.

S-layer extracts were then analyzed using antibodies raised against the specific repeat types I–V. Antibodies against repeat types I, II, IV and V recognized only their cognate strains 630, R20352, M9 and AY1, respectively. (Figure 4D). Anti-CwpVrptIII detects CwpV repeats in CDKK167, M9 and AY1 which all contain at least one type III repeat. Detection of CwpV in strain M9 with anti-CwpVrptIII is weak, this may be due to the presence of only one type III repeat in this CwpV protein. These results demonstrate that, as expected based on distinct amino acid sequence, the CwpV repeat types are antigenically distinct.

### Expression of all CwpV types is phase variable

**Figure 5:**
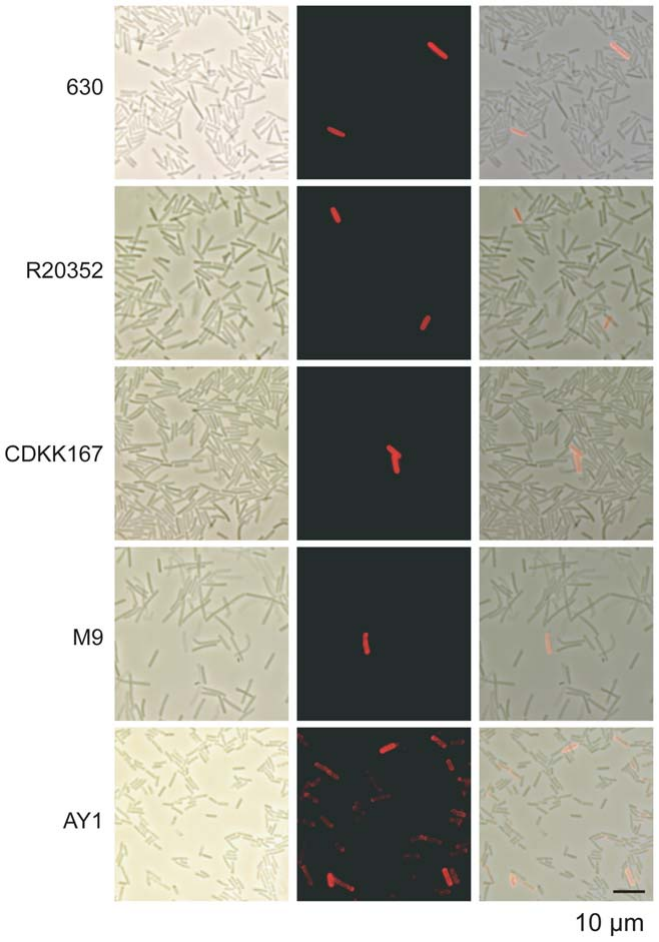
Expression of all CwpV types is phase variable. Overnight *C. difficile* cultures of each strain were stained with the appropriate anti-CwpVrpt antibody (630, rptI; R2035, rptII; CDKK167, rptIII; M9, rptIV; AY1, rptV) then with anti-rabbit rhodamine red-X. From left to right; phase image, fluorescent image, overlay.

Inversion of the *cwpV* switch has been detected in a wide variety of strains, but phase variable expression of CwpV has only been reported in strain 630 [19]. It therefore seemed possible that CwpV would be expressed in a phase variable manner in strains expressing all types of CwpV. Cultures of representative strains for each type were stained using the appropriate anti-CwpVrpt antibody for analysis by immunofluorescence microscopy (Figure 5). All strains expressed CwpV in a subset of cells, ranging from 0.1–10% of total cells, indicative of phase variable expression. The factors determining the proportion of CwpV-positive cells are yet to be determined, but it is clear that under standard laboratory culture conditions, a minority of cells express CwpV.

### A conserved post-translational cleavage mechanism for all CwpV proteins

**Figure 6:**
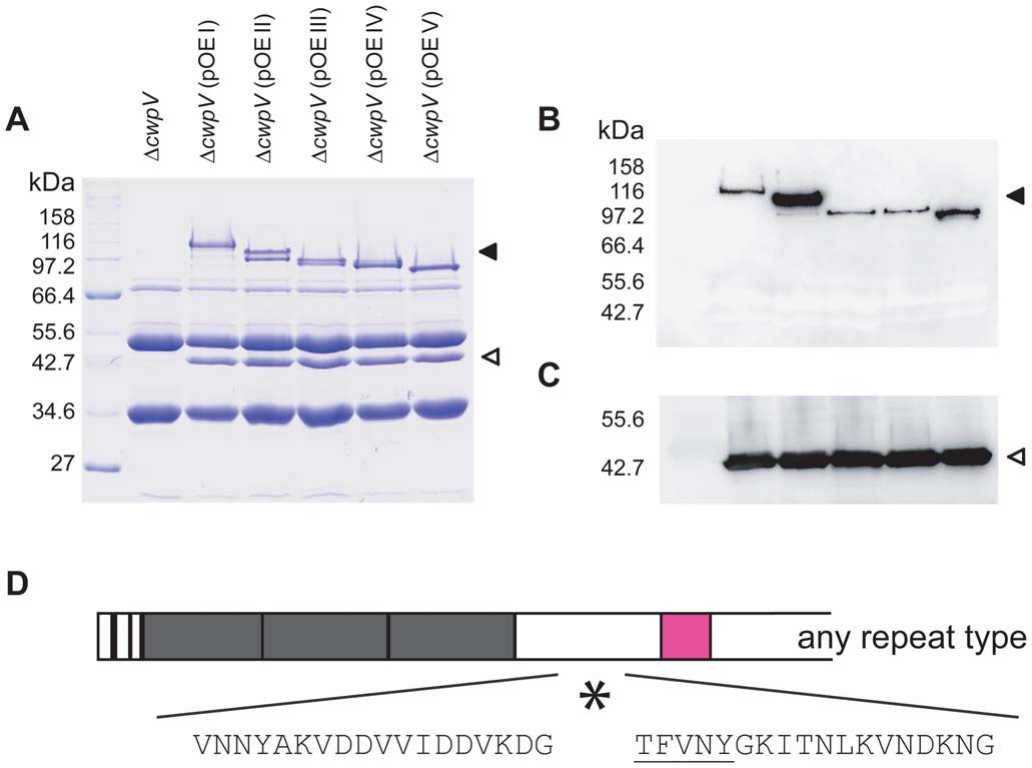
A conserved post-translational cleavage site for all CwpV proteins. **A.** S-layer extracts from 630Δ*cwpV* and 630Δ*cwpV* containing plasmids pOEI-V that over-express CwpV types I-V with a C-terminal Strep-tag were analysed by SDS-PAGE gel and Coomassie staining. ◂, repeat domain; ◃, N-terminal domain. **B.** Western blots of S-layer extracts using anti-Strep tag antibody detects the Strep-tagged CwpV C-terminal repeat domain. **C.** Western blots of S-layer extracts using anti-CwpVNter antibody detects the CwpV N-terminal domain. **D.** N-terminal sequencing of all strep-tagged CwpV C-termini yielded the sequence TFVNY, revealing the conserved cleavage site for all CwpV types. As illustrated, the cleavage site (*) is located between the cell wall anchoring domains (grey) and the serine/glycine-rich region (pink).

The five different CwpV proteins from 630, R20352, CDKK167, M9 and AY1 were modified to include a C-terminal Strep-Tag II and expressed in *C. difficile* 630Δ*cwpV* using a constitutive promoter. S-layer extracts were prepared from these strains, designated Δ*cwpV*(pOE I-V), and analyzed by SDS-PAGE (Figure 6A). A high level of CwpV expression can be seen for each CwpV protein, with CwpV constituting a significant proportion of the total S-layer extract. All five proteins are cleaved into the ∼42 kDa N-terminal fragment and C- terminal fragment of between 90 and 120 kDa, depending on their CwpV type. Western blotting using antibodies against the N-terminal domain detected the 42 kDa fragment in each extract and those against the Strep-Tag II detected the 90–120 kDa C-terminal CwpV fragment (Figure 6B). In order to determine the cleavage site for each CwpV protein, the N-terminal sequence of each strep-tagged C-terminal fragment was obtained. The first 5 amino acids of every C-terminal CwpV fragment was found to be TFVNY, unambiguously locating the cleavage site in the domain of CwpV between the N-terminal domain and the serine/glycine rich region, as depicted in Figure 6D. This domain is well-conserved between CwpV proteins of different types, and indicates that the CwpV post-translational cleavage mechanism is conserved across CwpV types. The protease responsible for SlpA cleavage has been identified as the surface expressed cysteine protease Cwp84 [8], [9] however it has been shown that Cwp84 is not required for CwpV cleavage [8]. We have analyzed expression of CwpV in strains containing knock-outs of either *cwp84* or its paralog *cwp13*, and found that neither protease is required for cleavage of CwpV (data not shown).

### All types of CwpV form a complex of the N-terminal and C-terminal fragments

**Figure 7:**
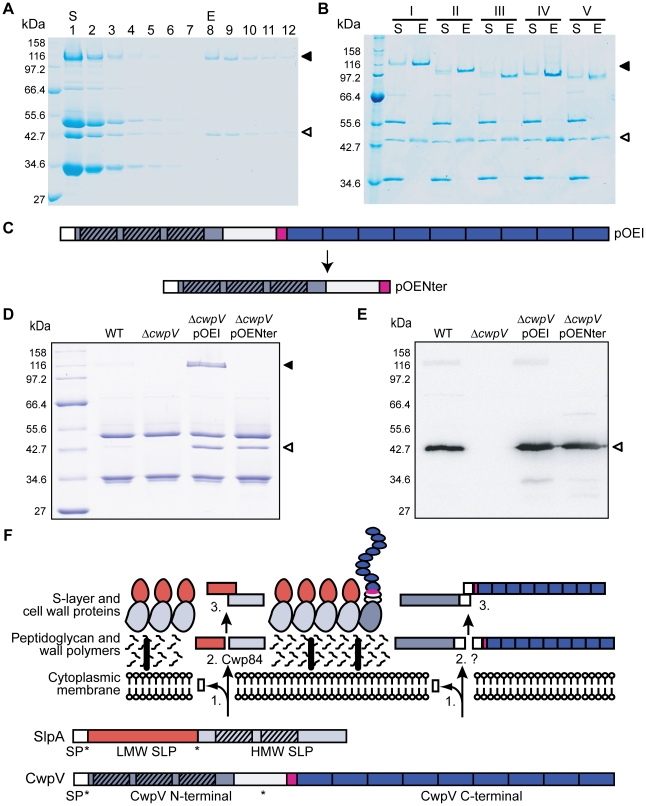
CwpV cleavage products form a complex that is anchored to the cell wall by the N-terminal domain. **A.** Streptactin binding/elution assay of a 630Δ*cwpV*(pOE I) S-layer extract analysed by SDS-PAGE. Lanes 1, S-layer extract (S); 2, Streptactin resin flow through; 3–7, washes; 8–12, elutions (E). The ∼42 kDa CwpV N-terminal fragment (◃) and ∼116 kDa CwpV C-terminal strep-tagged fragment (◂) are indicated. **B.** S-layer extracts (S) and first elution (E) from 630Δ*cwpV*(pOEI-V) streptactin binding/elution assays. In each lane E, the CwpV N-terminal (◃) and C-terminal (◂) fragments can be seen. **C.** Illustration of full length CwpV (pOEI, top) and the truncated version of CwpV (pOENter; bottom). **D.** Expression of truncated CwpV in Δ*cwpV* visualized by SDS-PAGE, compared to WT 630, 630Δ*cwpV* and 630Δ*cwpV*(pOEI). Truncation of CwpV does not affect the expression of the CwpV N-terminal fragment in the *C. difficile* S-layer. **E.** Western blot analysis of the S-layer extracts from 630, 630Δ*cwpV*, 630Δ*cwpV*(pOEI) and 630Δ*cwpV*(pOENter) using anti-CwpVNter antibody. **F.** Cartoon representation of the overall model for post-translational CwpV processing and incorporation into the S-layer with analogy to SlpA processing. +×, cleavage sites. Step 1, signal peptide (SP) cleavage and transport across the cell membrane. Step 2, cleavage of protein. SlpA is cleaved by Cwp84 into the LMW and HMW SLP. The protease responsible for CwpV cleavage is currently unknown. Step 3, formation of a complex of the products of cleavage, anchoring both products to the cell surface.

Post-translational cleavage of CwpV into two fragments is reminiscent of post-translational cleavage of SlpA into the HMW and LMW SLP fragments. In the case of SlpA these two fragments form a complex allowing the HMW SLP, containing the putative cell wall binding motifs, to anchor the LMW SLP to the underlying cell wall [10]. We therefore reasoned that CwpV cleavage may also be followed by complex formation to allow the N-terminal domain, containing putative cell wall binding motifs, to anchor the repetitive C-terminal fragment to the cell surface. To test this hypothesis, the C-terminal repeat region from strain Δ*cwpV*(pOEI) was purified by affinity chromatography using a StrepTactin resin. As shown in Figure 7A, the 42 kDa and 116 kDa fragments of CwpV were co-eluted despite the presence of the Strep-Tag II only in the larger fragment. This experiment was then repeated with S-layer extracts from Δ*cwpV* strains over-expressing CwpV types II–V. The complete S-layer extracts (S) and first elutions (E) are visualized by SDS-PAGE (Figure 7B). In all cases, both CwpV fragments co-eluted, clearly demonstrating complex formation. As this interaction is independent of repeat type, this strongly suggests that, as in SlpA, it is the highly conserved regions adjacent to the cleavage site that mediate complex formation.

### The CwpV N-terminal fragment is responsible for cell wall anchoring

The role of the N-terminal fragment of CwpV containing putative cell binding motifs was then investigated. A truncated form of the 630 *cwpV* gene which lacks the whole repetitive sequence downstream of the serine/glycine region was created as depicted in Figure 7C. The plasmid encoding this protein, pOE Nter, was transferred to 630Δ*cwpV* to create Δ*cwpV*(pOENter). S-layer extracts from wild-type, Δ*cwpV*, Δ*cwpV*(pOE I) and Δ*cwpV*(pOENter) cultures were analysed by SDS-PAGE (Figure 7D). The ∼42 kDa N-terminal CwpV fragment is seen in all extracts other than Δ*cwpV* and its identity was confirmed by Western blot with anti-CwpVNter (Figure 7E). This suggests that the N-terminal fragment of CwpV alone is sufficient for stable association with the cell wall. The same amount of the N-terminal CwpV fragment is seen in the Δ*cwpV*(pOEI) and Δ*cwpV*(pOENter) S-layer extracts, showing that the truncated version of CwpV is equally well expressed on the cell surface as the full-length CwpV protein. We therefore named the N-terminal fragment of CwpV as the cell wall anchoring (CWA) fragment. This is the first reported experimental evidence that PF04122 motifs are responsible for cell wall anchoring.

### A model for post-translational CwpV processing

The determination of the CwpV cleavage site, evidence of complex formation by the two CwpV cleavage products, and role of the N-terminal fragment in cell wall anchoring leads to an overall model for post-translational CwpV processing. The three steps involved are depicted in Figure 7F, and are compared to the steps known to occur for SlpA processing. The first step is export of the protein across the cell membrane and cleavage of the signal peptide. Once on the surface the protein is cleaved (step 2) by Cwp84 in the case of SlpA, or by an unknown mechanism in the case of CwpV. The third step is complex formation of the cleavage products, allowing the HMW SLP or the CwpV CWA fragment to anchor the complex to the cell surface. All three steps appear to be conserved amongst different CwpV types.

### CwpV is an aggregation-promoting factor

**Figure 8:**
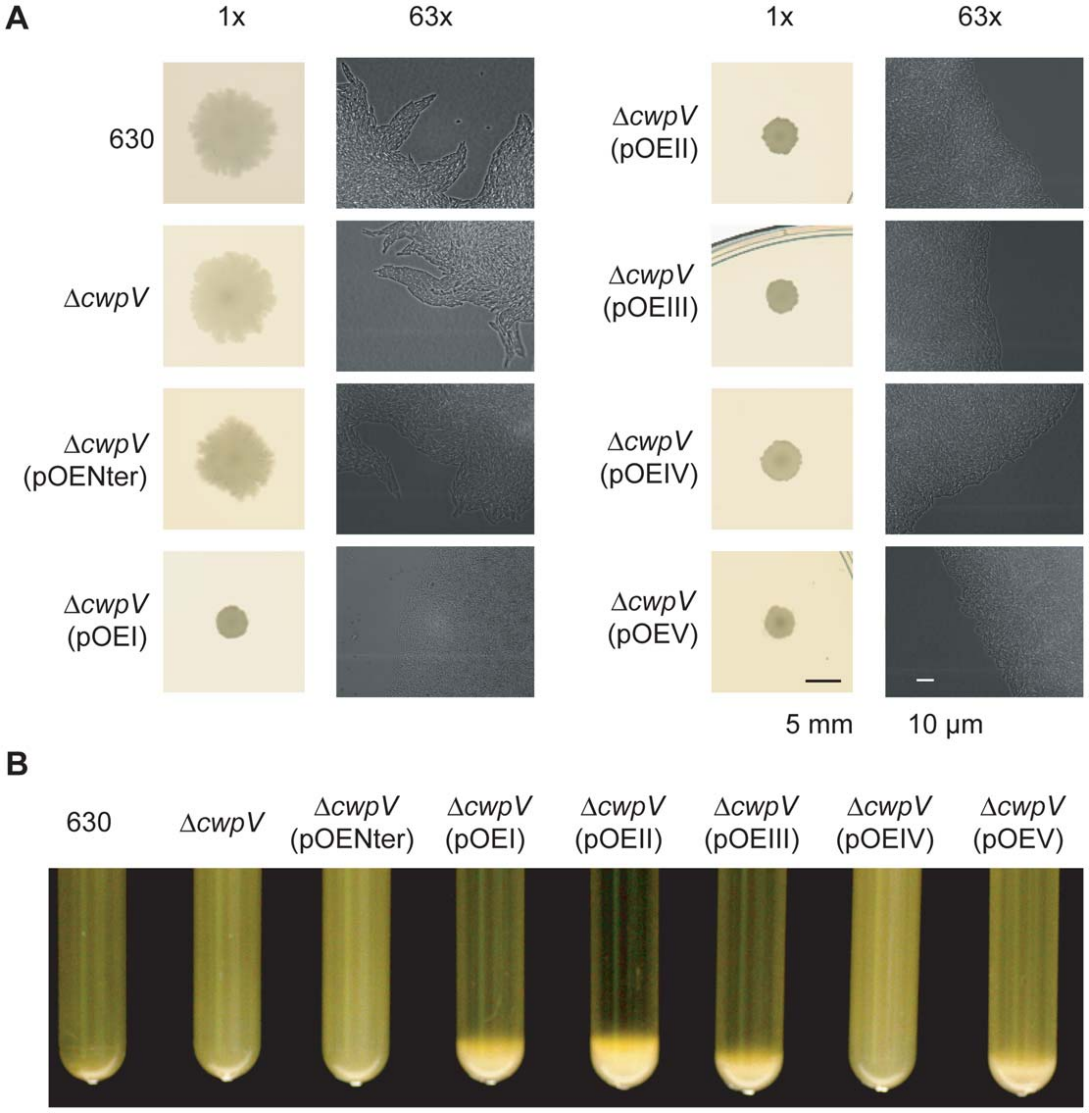
CwpV is an aggregation-promoting factor. **A.** Colony morphology caused by CwpV over-expression in *C. difficile*. 630, 630Δ*cwpV*, and 630Δ*cwpV*(pOENter) exhibit the same colony morphology. 630Δ*cwpV*(pOEI-V) all exhibit a smaller, smoother-edged colony morphology. These differences can be seen at the macroscopic level, shown by the 1× photographs on the left for each strain. Differences at the colony boundaries can be seen microscopically shown by the 63× images on the right for each strain. **B.** Aggregation of *C. difficile* in suspension (starting OD_600nm_ of 10) was observed in suspensions of Δ*cwpV*(pOEI, II, III and V), but not by 630, Δ*cwpV*, Δ*cwpV*(pOENter) or Δ*cwpV* (pOEIV).

As noted previously, Δ*recV* ON strains showed a smaller, smoother-edged colony morphology than Δ*recV* OFF or wild-type strains (see Figure 1D). We therefore investigated this phenomenon in greater detail. As shown in Figure 8A, wild-type and Δ*cwpV* strains exhibit the same disperse, ruffled colony morphology. Over-expression of the CwpV N-terminal CWA alone in Δ*cwpV* did not alter this colony morphology. However, over-expression of any of the five types of full-length CwpV caused the same smaller, smoother-edged colony morphology seen in Δ*recV* ON mutants (Figures 1D and 8A). These results strongly suggest that it is the repetitive C-terminal domain of CwpV that mediates this phenotype. In order to investigate more closely the basis of this difference, the edges of colonies grown between glass and agar were visualized microscopically. Detail of the colony morphology at the cellular level can be seen in the 63× magnification images shown alongside the 1× images in Figure 8A. Wild-type, Δ*cwpV* and Δ*cwpV*(pOENter) colonies were seen to have diffuse edges, with directional protrusions of bacterial growth common at the colony edge. However, Δ*cwpV*(pOEI-V) exhibited denser, straighter colony boundaries, with rare if any directional protrusions. The observed macroscopic differences in colony morphology are therefore due to altered cellular organization within bacterial colonies.

This altered cellular organization may result from CwpV-mediated auto-aggregation. Therefore the propensity of *C. difficile* to aggregate from suspension was assessed across the panel of strains. Over-expression of all CwpV types, except type IV, induced aggregation of *C. difficile* from a liquid suspension with a starting OD_600 nm_ of 10 (Figure 8B). Aggregation was followed in real time, and could be observed in tubes where aggregation was occurring from as soon as 1 hour after suspension of bacteria (data not shown). It was found that only 630 CwpV (type I) induced aggregation of *C. difficile* from suspensions with an OD_600 nm_ of 3, while 630 CwpV and R20352 CwpV (type II) induced aggregation from an OD_600 nm_ of 6 (data not shown). An OD_600 nm_ as high as 40 did not lead to type IV-mediated aggregation, nor aggregation of wild-type, Δ*cwpV* or Δ*cwpV*(pOENter) (data not shown). Therefore it appears that the ability to induce aggregation is highest for CwpV from 630, followed by R20352, then CDKK167 and AY1, with no observed propensity of M9 CwpV or the CwpV CWA fragment to induce aggregation of *C. difficile* from suspension.

## Discussion

We previously described the phase-variable expression of CwpV, employing a novel transcriptional termination mechanism [19]. Here we show that CwpV can constitute a major cell wall protein and that it exhibits an auto-aggregation promoting function. Surprisingly, this function is mediated by the highly variable C-terminal repeat domain which we find in five entirely distinct sequence types. Auto-aggregation was analyzed at both the cellular level on solid media and within liquid culture. *In vitro* colonies are organized populations of bacteria and differences in colony morphology reflect the cellular organization of a population [22] that can translate to important differences affecting natural growth dynamics. Differences in the cellular organization of colonies was clearly demonstrated by microscopy (Figure 8A) with CwpV expression in all cells causing a denser, more randomly packed cellular organization than the diffuse, directional patterns of cellular packing seen at the edge of wild-type colonies. The CwpV-mediated aggregation of *C. difficile* cells from suspension strongly suggests that CwpV has a direct aggregation-promoting function rather than a direct effect on motility. In fact the motility of strains in soft agar was found to be similar, regardless of whether or not they expressed CwpV (see Figure S2 and Text S1).

Propensities of different CwpV types to cause aggregation varied, as observed by the different starting densities of suspensions required for aggregation, apart from type IV, which did not induce aggregation. This is likely to reflect different affinities of the different CwpV proteins for their ligand(s). The lack of an observable aggregation phenotype due to type IV repeats may be explained by a weaker affinity between this repeat type and its ligand, however the observed colony morphology phenotype does suggest that it carries out the same general function as the other types. Functional interactions between CwpV and motility factors, such as type IV pili and flagella, are worthy of further study. Indeed, the diffuse directional organization of wild-type colony edges seen in this study is reminiscent of twitching motility mediated by type IV pili in *Clostridium perfringens* [23]. *C. difficile* does have type IV pili clusters, which are conserved across strains [24]. Type IV pili may therefore be involved in the cellular organization of wild-type colonies grown on solid surfaces observed here. Auto-aggregative proteins have been shown to play roles in biofilm formation [25], [26], [27]. No studies on *C. difficile* biofilms have been reported, and in our experience *C. difficile* does not exhibit significant biofilm formation on abiotic surfaces (data not shown). However in infected mice large aggregates of *C. difficile* cells, described as exaggerated mats, were reported to be associated with regions of severe inflammation [28]. This description is indicative of a biofilm-like growth of *C. difficile* during infection. Multispecies biofilms in the human GI tract have been observed [29] and *C. difficile* is likely to require factors that promote appropriate intra- and inter-species interactions to colonize the gut. CwpV may play important roles in this process.

Aside from its aggregation-promoting function CwpV may have other functions. We investigated bacterial adhesion to Caco-2 cells, but no significant difference in adhesion was caused by CwpV expression (data not shown). Further work is required to assess whether CwpV expression affects colonization levels *in vivo*, and if so whether this is directly due to its aggregation-promoting activity. Understanding the roles of CwpV during *in vivo* infection is a priority and will be addressed using the isogenic mutants described in this study. Established animal models of infection focus more on the acute stage of *C. difficile* infection, rather than colonization [30]. However, recent developments of animal models better suited to studying the roles of *C. difficile* genes likely to be involved in colonization, such as CwpV, have been described [28], [31] that will facilitate our future investigation. The phase-variable expression of CwpV may be highly relevant to any *in vivo* role; its expression in a sub-set of cells as a contingency may be beneficial to the survival of the population as a whole.

The variation of CwpV repeat sequences uncovered in this study is extensive, with strains expressing one or two of five antigenically distinct types encoded by unrelated sequences. The lack of sequence homology between repeat types suggests acquisition by horizontal gene transfer. Any known mechanism of chromosomal sequence acquisition; homologous recombination, illegitimate recombination or additive integration could account for introduction of these sequences to the *C. difficile* chromosome [32]. However, without knowing the origin of these sequences, the mechanisms of transfer to *C. difficile* and incorporation into the genome are unclear. Human microbiome sequencing may shed light on the origins of CwpV sequences. It would seem that CwpV type exchange is occurring at a low frequency, as all strains tested from within one ribotype contain the same CwpV type. Current understanding of the phylogenetics and diversity of *C. difficile* strains is limited [21], [33]. However current data suggests a single clade is associated with one CwpV type; the HY (027) clade associates with type II CwpV, the A-B+ clade with type IV CwpV, the HA2 (078) and HA1 clades with type I CwpV. The locations of strains expressing type III and V CwpV within this phylogenetic tree of *C. difficile* are not yet known. As more *C. difficile* strains are sequenced and our understanding of evolutionary relationships between strains improves, it will become clearer as to when different CwpV types were acquired, and whether single or multiple independent acquisition events for each type have occurred. Host immune pressure is one possible selection that could promote CwpV variability, as one key difference between CwpV types is their antigenicity. Antigenic variability is also seen in the *C. difficile* surface proteins SlpA and Cwp66 [34]. Variation in surface proteins may also be involved in competition between different *C. difficile* strains or in resistance to bacteriophages.

Apart from the marked diversity in CwpV repeat sequences, other aspects of CwpV are remarkably conserved. Expression of all types of CwpV is phase variable suggesting that this complex regulatory mechanism, involving the conserved *cwpV* switch and the RecV recombinase, is important for optimal function of CwpV. Phase variation of bacterial surface proteins has been reported to be involved in numerous processes including immune evasion and colonization [35]. Given the sequence variability and aggregation-promoting function of CwpV, several explanations behind its phase variable regulation can be suggested. However, investigation into CwpV expression during *in vivo* infection is likely to shed most light on the importance of CwpV phase variability.

Conserved post-translational processing of CwpV and formation of a CWA-repeat domain complex again suggests that these processes are important for optimal CwpV expression and function. To date we only observe complex formation in cell wall proteins CwpV and SlpA, with all other CWPs studied (e.g. Cwp84, Cwp66, Cwp2) being presented on the surface without post-translational cleavage between the CWA and functional domains. An unidentified protease could mediate cleavage of CwpV, or it could undergo non-enzymatic auto-processing as reported for a number of protein families [36], [37]. Uncleaved SlpA is poorly tolerated on the cell wall, and is found in large quantities in the culture supernatant of a Δ*cwp84* mutant [9]. SlpA monomers once cleaved must interact on the cell surface to form a two-dimensional array. Expression of CwpV does not appear to disrupt the integrity of the S-layer and, by analogy, it seems likely that cleavage of CwpV is important for its stable incorporation into the S-layer. Given the high proportion of the total S-layer accounted for by CwpV in Δ*recV* ON strains, it seems likely that CwpV interacts positively with other S-layer proteins to maintain the integrity of S-layer packing.

Our finding that the CwpV CWA domain is responsible for anchoring of the CwpV C-terminal domain to the underlying cell wall is the first direct evidence of the function of Pf04122 domains. These domains are also present in the HMW SLP of the *C. difficile* S-layer where the presumably mediate a similar role. They are also found in a large variety of Gram-positive bacteria and some Archaea, where they presumably fulfill a similar function of anchoring cell wall proteins to the underlying structure. This then adds to the diversity of mechanisms used by Gram-positive bacteria to anchor and display cell wall proteins [38], [39].

Phylogenetic studies [21] show that outbreak-causing strains of *C. difficile* have emerged independently from multiple lineages, indicating that virulent strains can emerge from across the diversity of the species, rather than being confined to a specific pathogenic lineage. This suggests that certain genetic elements common to all *C. difficile* strains underlie virulence. It is likely that the incidences of different lineages causing disease are dynamic, and we cannot predict which lineages may cause future outbreaks. Therefore understanding the common features of *C. difficile* as an ancient species is important, as in order for intervention strategies to be successful in the long-term they must target the full diversity of *C. difficile* strains, rather than specific lineages. CwpV appears to be a core component of *C. difficile*, including its phase variable regulation controlled by RecV, post-translational processing mechanism, and aggregation-promoting function. This highlights the importance of CwpV to *C. difficile* survival throughout evolution of this diverse species. Despite this conservation CwpV appears to have been under positive selection for antigenic variability. Further investigation into the roles of CwpV in the *C. difficile* life cycle may teach us a lot about what makes *C. difficile* a successful pathogen, and provide opportunities for new intervention strategies.

## Materials and Methods

### Bacterial strains and growth conditions

The *C. difficile* strains characterised for CwpV type in this study are described in Table 1. *C. difficile* laboratory-generated mutant and recombinant-plasmid containing strains used in this study are described in Table S3. *C. difficile* was routinely cultured on blood agar base II (Oxoid) supplemented with 7% horse blood (TCS Biosciences), brain-heart infusion (BHI) agar (Oxoid) or in BHI broth (Oxoid). Cultures were grown in an anaerobic cabinet (Don Whitley Scientific) at 37°C in an atmosphere of 10% CO_2_, 10% H_2_ and 80% N_2_. *C. difficile* strains containing recombinant plasmids were grown under thiamphenicol selection. Commercial chemically competent TOP10 *E. coli* cells (Invitrogen) were used for cloning and recombinant plasmid maintenance. Recombinant expression of CwpV fragments was carried out in Rosetta *E. coli* cells (Novagen). *E. coli* strains were routinely grown at 37°C on LB-agar plates or in LB liquid culture in the presence of selective antibiotics where appropriate to any transformed plasmid.

### DNA manipulations

DNA manipulations were carried out according to standard techniques. *C. difficile* genomic DNA for use in cloning and PCR analysis was prepared as described previously [19]. Polymerase chain reactions (PCR) used KOD (Novagen), Expand Long-Template Polymerase (Roche Diagnostics) or Taq polymerase (Sigma) in accordance with the manufacturers' protocols using primers detailed in Table S1.

### Production of recombinant plasmids

In order to produce a *C. difficile* 630Δ*erm* knock out of *recV*, a retargeted ClosTron vector was designed using the intron design tool www.clostron.com. The top-scored target site in *recV* was selected (424/425s) and the intron targeting region required was commercially synthesized and cloned into pMTL007C-E2 (DNA 2.0) to generate a plasmid pLRP028. For complementation of the Δ*recV* mutant, the *recV* gene from *C. difficile* 630 was amplified using primers NF1357 and NF1358 and cloned using *Sac*I and *Bam*HI into a *C. difficile* plasmid expression vector, based on the pMTL960 replicon [19], [40] to generate pCBR113 (pRecV+). Inverse PCR with primers NF1411 and NF1412, using pCBR113 as the template, was carried out to mutate the *recV* tyrosine176 to phenylalanine, producing pCBR115 (pRecV_Y176F_+). To express CwpV fragments for use as antigens (Figure 4A) *cwpV* repeats from strains R20352 (type II), CDKK167 (type III), M9 (type IV) and AY1 (type V) were amplified by PCR using KOD polymerase (Novagen/Merck Chemicals Ltd) and primers described in Table S1. These fragments were directionally cloned using *Nco*I and *Xho*I into pET28a *E. coli* expression vector then transformed into TOP10 *E. coli* (Invitrogen) to generate plasmids pCBR069-072 described in Table S2. To clone the *cwpV* genes from strains of unknown genomic sequence, genomic DNA was digested overnight at 37°C with *Ase*I (NEB) and then ligated overnight at 16°C using T4 ligase (NEB). Inverse PCR using primers NF401 and NF654 was carried out and amplification products were cloned into pCR4-TOPO (Invitrogen). pCBR044, containing the full-length *cwpV* gene from 630 under the control of the *cwp2* promoter within the pMTL960 backbone [19], was modified to contain a 5′ XhoI site and strep-tag II (WSHPQFEK) encoding sequence by inverse PCR, generating the *C. difficile* strep-tagged 630 CwpV expression plasmid pCBR080 (pOEI). The *cwpV* genes from R20352, CDKK167, M9 and AY1 were amplified with the primers described in Table S1 and cloned in place of the 630 gene in pCBR080 to create plasmids pCBR105-107 and 109 (pOEII-V) (Table S2). Successful construction of recombinant plasmids was always confirmed by DNA sequencing (GATC Biotech).

### Conjugation into *C. difficile*

Plasmids were transformed into *E. coli* CA434 and then conjugated into *C. difficile* as described previously [40] using thiamphenicol (30 mg ml^−1^) to select for pMTL960-based plasmids and cycloserine (250 mg ml^−1^) to counter-select for *E. coli*.

### Purification of recombinant CwpV protein and generation of antibodies

Plasmids pCBR069-072 expressing repeat types II–V were transformed into *E. coli* Rosetta (Novagen/Merck Chemicals Ltd) and grown overnight at 37°C in Overnight Express media (Novagen/Merck Chemicals Ltd). Bacteria were collected, lysed using BugBuster (Novagen/Merck Chemicals Ltd) and the His-tagged fusion proteins purified by affinity chromatography using Ni-NTA agarose (Qiagen). Following purification, proteins were dialysed into 10 mM HEPES 150 mM NaCl. Antisera to the proteins were generated in rabbits using a commercial service (Covalab, Cambridge, UK). Anti-CwpVNter and anti-CwpVrptI were described previously [19].

### S-layer extraction, SDS-PAGE and western immunoblotting

*C. difficile* cultures were grown overnight, harvested by centrifugation and S-layer extracts were prepared using low pH glycine incubation as described previously [7]. Proteins in the S-layer extracts were subjected to SDS-PAGE and western blotting according to standard protocols. All rabbit primary anti-CwpV antibodies (anti-CwpVNter, anti-CwpVrptI, anti-CwpVrptII, anti-CwpVrptIII, anti-CwpVrptIV and anti- CwpVrptV) were used at 1/5000 dilution, followed by anti-rabbit-HRP (Dako Cytomation) at 1/2000 dilution. Signal was detected using SuperSignal® West Pico Chemiluminescent Substrate (Pierce).

### Immunocytochemistry

*C. difficile* cells from liquid culture were washed with PBS then fixed in 8% formaldehyde, which was quenched with 20 mM NH_4_Cl for 15 min prior to staining. Washed cell suspensions were re-suspended in 40 µl 1% BSA in PBS containing 1/20 dilutions of rabbit primary antibodies (anti-CwpVNter, anti-CwpVrptI, anti-CwpVrptII, anti-CwpVrptIII, anti-CwpVrptIV and anti- CwpVrptV) for 45 min. Bacteria were then washed and incubated with 1/40 anti-rabbit-rhodamine red-X (Jackson ImmunoResearch) in the dark for 45 min. Bacteria were washed with PBS and dH_2_0 then allowed to air dry onto a microscope slide. Coverslips were mounted using ProLong gold anti-fade mounting reagent (Invitrogen) and allowed to set overnight. Bacteria were visualised using a Nikon Eclipse E600 microscope fitted with a Nikon DMX1200 camera.

### N-terminal sequencing

S-layer extracts were separated on 10% SDS-PAGE gels and transferred to a PVDF membrane. The membrane was washed in water for 10 min, stained in 0.1% Coomassie Blue R in 50% MeOH for 5 min. After destaining in 50% MeOH, 10% acetic acid (3×5 min with rocking) and washing in water (3×5 min with rocking) the membrane was allowed to dry thoroughly. N-terminal sequencing by Edman degradation was then carried (PNAC Facility, Department of Biochemistry, University of Cambridge).

### *C. difficile* S-layer extract streptactin binding/elution assay

*Strep*Tactin resin (Novagen) was used according to manufacturers' instructions. Briefly, resin was washed with dH_2_O, equilibrated with wash buffer (150 mM NaCl, 100 mM Tris-HCl, 1 mM EDTA, pH 8.0) and mixed with S-layer extract. The mixture was incubated overnight at 4°C with rotation. The next day the resin was washed with wash buffer to remove non-specifically bound proteins. Elution buffer (150 mM NaCl, 100 mM Tris-HCl, 1 mM EDTA, 2.5 mM desthiobiotin, pH 8.0) was then used to elute strep-tagged proteins. All fractions were collected for analysis using SDS-PAGE and Coomassie staining.

### Visualization of *C. difficile* colony morphology

For macroscopic visualization of *C. difficile* colony morphology strains were grown on BHI agar (Oxoid) for 5 days from an appropriate *C. difficile* inoculum such that isolated colonies grew on the plate. Plates were then scanned using a flat-bed scanner to record images of the colonies. For microscopic visualization of *C. difficile* colony morphology, cultures taken from a 24-hour old colony were inoculated directly on to glass-bottomed dishes (MatTek corporation). Anaerobic BHI 0.7% agar at 40°C was then carefully poured over the glass to cover the dish and allowed to set. *C. difficile* was grown between the glass and the agar for 3 days. Dishes were removed and images taken using a Zeiss Axiovert 200 widefield microscope at FILM, Imperial College London. Microscope images were analysed using Volocity 5.3.2 software (Improvision).

## Supporting Information

Figure S1 Sequence alignments of the C-terminal repeats in CwpV types I–V. (A) The nine type I repeats in strain 630 CwpV. (B) The eight type II repeats in strain R20352 CwpV. (C) The six type III repeats in strain CDKK167 CwpV. (D) *C. difficile* strains M9 and AY1 encode CwpV with mosaics of type III and type IV or V repeats. A cartoon representations of these mosaic CwpV proteins is shown above a ClustalW2 multiple sequence alignment of all type III repeats. (E) The seven type IV repeats in strain M9 CwpV. (F) The five type V repeats in strain of AY1 CwpV. The sequences of all repeats with a type were aligned using ClustalW2 and are shown in color, corresponding to the blocks in the cartoon, with the exception of type II repeats which are shown in black instead of yellow.
(PDF)

Figure S2 CwpV expression in *C. difficile* does not affect flagellar expression or swimming motility. (A) *C. difficile* strains were analyzed for FliC expression based on a published protocol [1]. *C. difficile* FliC expression from the panel of strains with varying levels of CwpV expression was assessed by SDS-PAGE and Coomassie staining, with 630 FliC running at ∼33 kDa. Lanes 1: WT, 2: Δ*cwpV*, 3: pOE Nter, 4–8: pOE I-V, 9: Δ*recVON*, 10: Δ*recVOFF*. (B) To assess swimming motility of *C. difficile* strains, BHI containing 0.175% agar was inoculated to a defined depth with *C. difficile* from overnight liquid cultures. Tubes were incubated overnight then photographed to document motility. Tubes are numbered in the same way as the lanes in A. All cultures appeared to be equally motile.
(TIF)

Table S1 Primers used in this study.
(DOC)

Table S2 Plasmids used in this study.
(DOC)

Table S3 Genetically modified *C. difficile* strains used in this study.
(DOC)

Text S1 References for Figure S2 and Tables S2 and S3.
(RTF)
