# Supplementary material for: The Clostridium difficile Cell Wall Protein CwpV is Antigenically Variable between Strains, but Exhibits Conserved Aggregation-Promoting Function
Source: PLoS Pathog. 2011 Apr 21;7(4):e1002024. doi: 10.1371/journal.ppat.1002024 (PMC3080850; doi:10.1371/journal.ppat.1002024)
Supplement: Figure S1 — Sequence alignments of the C-terminal repeats in CwpV types I–V. (A) The nine type I repeats in strain 630 CwpV. (B) The eight type II repeats in strain R20352 CwpV. (C) The six type III repeats in strain CDKK167 CwpV. (D) C. difficile strains M9 and AY1 encode CwpV with mosaics of type III and type IV or V repeats. A cartoon representations of these mosaic CwpV proteins is shown above a ClustalW2 multiple sequence alignment of all type III repeats. (E) The seven type IV repeats in strain M9 CwpV. (F) The five type V repeats in strain of AY1 CwpV. The sequences of all repeats with a type were aligned using ClustalW2 and are shown in color, corresponding to the blocks in the cartoon, with the exception of type II repeats which are shown in black instead of yellow. (PDF) [file ppat.1002024.s001.pdf]

A

630 : Type I

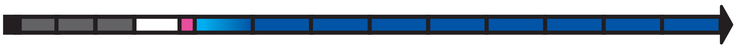

```
rpt6 GQLTFDATDKQIKMASGDKTVDPSDDTYVLTLTKGTAKDGDVKANVEVTGLPSGLDYTAE 60
rpt7 GQLTFDATDKQIKMASGDKTVDPSDDTYVLTLTKGTAKDGDVKANVEVTGLPSGLDYTAE 60
rpt5 GQLTFDATDKQIKMASGDKTVDPSDDTYVLTLTKGTAKDGDVKANVEVTGLPSGLDYTAE 60
rpt4 GQLTFDATDKQIKMASGDKTVDPSDDTYVLTLTKGTAKDGDVKANVEVTGLPSGLDYTAE 60
rpt2 GQLTFDATDKQIKMASGDKTVDPSDDTYVLTLTKGTAKDGDVKANVEVTGLPSGLDYTAE 60
rpt3 GQLTFDATDKQIKMASGDKTVDPSDDTYVLTLTKGTAKDGDIKANVEVTGLPSGLDYTAE 60
rpt9 GQLTFDATDKQIKMASGDKTVDPSDDTYVLTLTKGTAKDGDVKANVEVIGLPSGLDYTAE 60
rpt8 GQLTFDATDKQIKMASGDKTVDPSDDTYVLTLTKGTAKDGDVKANVEVIGLPSGLDYTAE 60
rpt1 GQIKFSATDKQIKMASGDKTVDPSDNTYILKLTNGTVKDGDVKANVKVAGLPSGLDYTAV 60
     **:.*.*******************:**:*.**:**.****:****:* **********

rpt6 GDSSANTITITVSGTASQAVQTDLDNVSVLVKAGAVSETTATDSAANATFEVKKYVAPAI 120
rpt7 GDSSANTITITVSGTASQAVQTDLDNVSVLVKAGAVSETTATDSAANATFEVKKYVAPAI 120
rpt5 GDSSANTITITVSGTASQAVQTDLDNVSVLVKAGAVSETTATDSAANATFEVKKYVAPAI 120
rpt4 GDSSANTITITVSGTASQAVQTDLDNVSVLVKAGAVSETTATDSAANATFEVKKYVAPAI 120
rpt2 GDSSTNTITITVSRTASQAVQTDLDNVSVLVKAGAVSETTATDSAANATFEVKKYVAAPA 120
rpt3 GDSSANTITITVSGTASQAVQTDLDNVSVLVKAGAVSETTATDSAANATFEVKKYVAAPA 120
rpt9 GDSSANTITITVSGTASQAVQTDLDNISVLVKAGAVSETTATDSAANATFEVKKHASDPI 120
rpt8 GDSSANTITITVSGTASQAVQTDLDNVSVLVKAGAVSETTATDSAANATFEVKKYVAPAI 120
rpt1 GNRSANTITITVSGTASQSIQNDLNNVSVLVKAGAVSGTGATDSIANTTFEIKKYVAPAI 120
     *: *:******** ****::*.**:*:********** * **** **:***:**:.:  .
```

B

R20352 : Type II

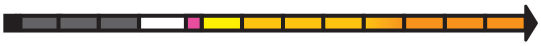

```
rpt2 AVDKTALQDAVTAATALHAKATEGTAEGNYAVGSKATYKTAIDEAQAILDKSDATQKEVN 60
rpt3 AVDKTALQDAVTAATALHNGATEGTAEGNYAVGSKATYKTAIDEAQAILDKSDATQKEVN 60
rpt4 AVDKTALQDAVTAATALHNGATEGTAEGEYAVGSKATYKTAIDEAQAILDKSDATQKEVN 60
rpt6 -LNKTALQDAVTAATSLHAGATEGTAEGNYAVGSKATYKTAIDEAQAILDKTGATQKEID 59
rpt7 -LNKTALQDAVTAATSLHAGATEGTAEGNYAVGSKATYKTAIDEAQAILDKTGATQKEID 59
rpt8 -LNKTALQDAVTAATSLHAGATEGTAEGNYAVGSKATYKTAIDEAQAILDKADATQKEID 59
rpt5 AVDKTALQDAVTAATALHNGATEGTAEGNYAVGSKATYKTAIDEAQAILDKTGATQKEID 60
rpt1 KVDKVVLKNTITAANKLYNEAIEGTNVGEYKVGSKAIYKTAIDKAQAILDKSGVTQKEVN 60
      ::*..*::::***. *:  * ***  *:* ***** ******:*******:..****::

rpt2 DALSALNTATETFEAGKVV------------------ 79
rpt3 DALSALNTATETFEAGKVV------------------ 79
rpt4 DALTALNTATETFEAGKVV------------------ 79
rpt6 DALSALNTATDTFKAGKVV------------------ 78
rpt7 DALSALNTATDTFKAGKVV------------------ 78
rpt8 DAVTALNTATATFEAGKVPTTIALMLSRILGFMKLEA 96
rpt5 DALSALNTATDTFKAGKVV------------------ 79
rpt1 DAVTALNTATDTFKAGKVV------------------ 79
     **::****** **:****
```

C

CDKK167 : Type III

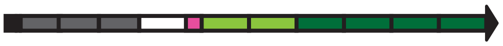

```
rpt4 RTAIDTEQLAVQAEADKITSVTQPTQDATTLTMPTVASGYTIKIKSSTNESVIAKDGTIV 60
rpt6 RTAIDTEQLAVQAEADKITSVTQPTQDATTLTMPTVASGYTIKIKSSTNESVIAKDGTIV 60
rpt5 RTAIDTEQLAVQAEADKITSVTQPTQDATTLTMPTVASGYTIKIKSSTNESVIAKDGTIV 60
rpt3 KSTDGEIQAEVQAEADKITSVTQPTQDATTLTMPTVASGYTIKIKSSTNESVIAKDGTIV 60
rpt1 ------NQTSVNNEAAKITSVPTPAKDATRLTMPSVSSGYTIAIKTSSNESVIKKDGTII 54
rpt2 KSTDEELQAALDNEVAKITSVPAPAKDATKLTMPSVSSGYTIAIKTSSNKSVIKEDGTII 60
            *  :: *. *****. *::*** ****:*:***** **:*:*:*** :****:

rpt4 PPDTETKVTLVFTVTHTSSGKIADTGEIEVTVPA------------------------ 94
rpt6 PPDTETKVTLVFTVTHTSSGKTADTGEIEVTVPARTTTVSSLLGRIAVNIFNFNKLEA 118
rpt5 PPDTETKVTLVFTVTHTSSGKTADTGEIEVTVPA------------------------ 94
rpt3 PPDTETKVTLVFTVTHTSSGKTADTGEIEVTVPA------------------------ 94
rpt1 PPNTATTVKLVFTVTHTSSGKTADTKELSVTVPA------------------------ 88
rpt2 PPNTEETVTLVFTVTQESSGKTADTGEIDVVVPA------------------------ 94
     **:*  .*.******: **** *** *:.*.***
```

D

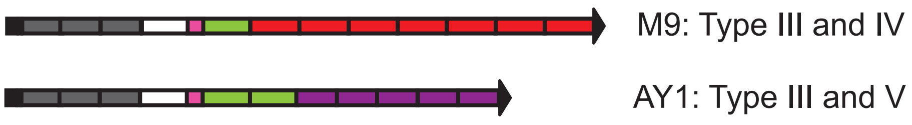


```
167rpt4  RTAIDTEQLAVQAEADKITSVTQPTQDATTLTMPTVASGYTIKIKSSTNESVIAKDGTIV 60
167rpt6  RTAIDTEQLAVQAEADKITSVTQPTQDATTLTMPTVASGYTIKIKSSTNESVIAKDGTIV 60
167rpt5  RTAIDTEQLAVQAEADKITSVTQPTQDATTLTMPTVASGYTIKIKSSTNESVIAKDGTIV 60
167rpt3  KSTDGEIQAEVQAEADKITSVTQPTQDATTLTMPTVASGYTIKIKSSTNESVIAKDGTIV 60
167rpt1  ------NQTSVNNEAAKITSVPTPAKDATRLTMPSVSSGYTIAIKTSSNESVIKKDGTII 54
AY1rpt1  ------NQTSVNNEAAKITSVPTPAKDATRLTMPSVSSGYTIAIKTSSNESVIKKDGTII 54
M9rpt1   ------NQTSVNNEAAKITSVPTPAKDATKLTMPSVSSGYSIAIKTSSNESVIKKDGTII 54
167rpt2  KSTDEELQAALDNEVAKITSVPAPAKDATKLTMPSVSSGYTIAIKTSSNKSVIKEDGTII 60
AY1rpt2  KSTDEELQAALDNEVAKITSVPAPAKDATKLTMPSVSSGYTIAIKTSSNKSVIKEDGTII 60
                   *  :: *. *****. *::*** ****:*:***:* **:*:*:*** :****:

167rpt4  PPDTETKVTLVFTVTHTSSGKIADTGEIEVTVPA------------------------ 94
167rpt6  PPDTETKVTLVFTVTHTSSGKTADTGEIEVTVPARTTTVSSLLGRIAVNIFNFNKLEA 118
167rpt5  PPDTETKVTLVFTVTHTSSGKTADTGEIEVTVPA------------------------ 94
167rpt3  PPDTETKVTLVFTVTHTSSGKTADTGEIEVTVPA------------------------ 94
167rpt1  PPNTATTVKLVFTVTHTSSGKTADTKELSVTVPA------------------------ 88
AY1rpt1  PPNTATTVKLVFTVTHTSSGKTADTKELSVTVPA------------------------ 88
M9rpt    PPNTATTVKLVFTVTHTSSGKTADTKELSVTVPA------------------------ 88
167rpt2  PPNTEETVTLVFTVTQESSGKTADTGEIDVVVPA------------------------ 98
AY1rpt2  PPNTEETVTLVFTVTQESSGKTADTGEIDVVVPAKSTK-------------------- 98
         **:*  .*.******: **** *** *:.*.***
```

E

```
M9typeIVrpt6  --KDVVAAVSTVNATNGTLTIVLDKTPTVDPVEGDFTAKKSIDGGQESELTLSNFAYNKE 58
M9typeIVrpt7  --KDVVAAVSTVNATNGTLTIVLDKTPTVDPVEGDFTAKKSIDGGQESELTLSNFAYNKE 58
M9typeIVrpt5  --KDVVAAVSTVNATNGTLTIVLDKTPTVDPVEGDFTAKKSIDGGQESELTLSNFAYNKE 58
M9typeIVrpt4  KGQDVVAAVSTVNATNGTLTIVLDKTPTVDPVEGDFTAKKSIDGGQESELTLSNFAYNKE 60
M9typeIVrpt2  KGQDVVAAVSTVNATNGTLTIVLDKTPTVDPVEGDFTAKKSIDGGQESELTLSNFAYNKE 60
M9typeIVrpt3  KGQDVVAAVATVNATNGTLTIVLDKTPTVDPVEGDFTAKKSIDGGQESELTLSNFVYNKE 60
M9typeIVrpt1  KSTEVQAAVSTVNATNGTLTIVLDKTPTVDPVEGDFTAKKSIDGGQESELTLSNFAYNKE 60
                 :*  ***:***************************************.****

M9typeIVrpt6  SKTVTYSFVPIEQTELEQSVVVGVDYKGENTKAAAFKVDA------------------------ 98
M9typeIVrpt7  SKTVTYSFVPIEQTELEQSVVIGVDYKGENTKAAAFIVNGNSVPTRTLKTNLTNVAKSVINLEA 122
M9typeIVrpt5  SKTVTYSFVPIEQTELEQSVVVGVDYKGENTKAAAFKVDA------------------------ 98
M9typeIVrpt4  SKTVTYSFVPIEQTELEQSVVVGVDYKGENTKAAAFKVDA------------------------ 100
M9typeIVrpt2  SKTVTYSFAPIEQTELEQSVVVGVDYKGENTKAAAFKVEA------------------------ 100
M9typeIVrpt3  SKTVTYSFTPIEQTELEQSVVVGVDYKGENTKAAAFKVEA------------------------ 100
M9typeIVrpt1  SKTVTYSFTPIEQTELEQSVVVGVDYKGENTKAAAFKVEA------------------------ 100
              ********.************:************** *:.
```

F

```
AY1typeVrpt3  VIIGEGSVSGATAGDKEITGLTSGKIYKVTMDGVEKYTKADGTLGEEGEKAALTGTSITG 60
AY1typeVrpt4  VIIGEGSVSGATAGDKEITGLTSGKIYKVTMDGVEKYTKADGTLGEEGEKAALTGTSITG 60
AY1typeVrpt1  VIIGEGSVSGATAGDKEITGLTSGKIYKVTMDGVEKYTKADGTLGEEGEKAALTGTSITG 60
AY1typeVrpt2  VIIGEGSVSGATAGDKEITGLTSGKIYKVTMDGVEKYTRADGTLGEEGEKAALTGTSITG 60
AY1typeVrpt5  VIIGEGSVSGATAGDKEITGLTSGKIYKVTMDGVEKYTKADGTLGEEGEKAALTGTSITG 60
              **************************************:*********************

AY1typeVrpt3  LVNGKTYKVEEYVAPVVNE--------------- 79
AY1typeVrpt4  LVNGKTYKVEEYVAPVVNE--------------- 79
AY1typeVrpt1  LVNGKTYKVEEYVAPVVNE--------------- 79
AY1typeVrpt2  LVNGKTYKVEEYVAPVVNE--------------- 79
AY1typeVrpt5  LVNGKTYKVEEDLSAISTIGRLMLKFTNLVKLEA 94
              *********** ::.: .
```
