# Supplementary figures and images for: The Clostridium difficile Cell Wall Protein CwpV is Antigenically Variable between Strains, but Exhibits Conserved Aggregation-Promoting Function
Source: PLoS Pathog. 2011 Apr 21;7(4):e1002024. doi: 10.1371/journal.ppat.1002024 (PMC3080850; doi:10.1371/journal.ppat.1002024)

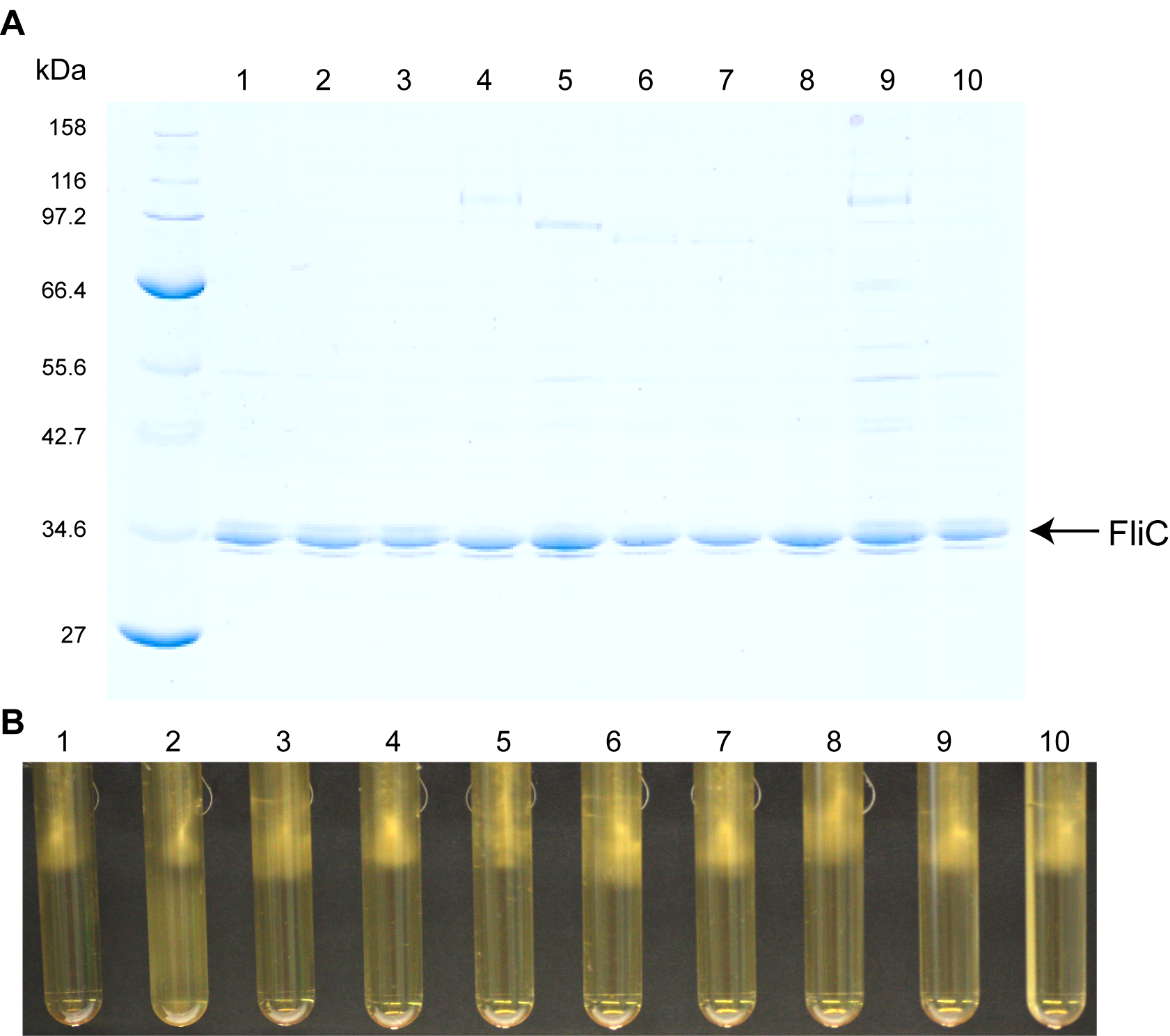

Supplement: Figure S2 — CwpV expression in C. difficile does not affect flagellar expression or swimming motility. (A) C. difficile strains were analyzed for FliC expression based on a published protocol [1]. C. difficile FliC expression from the panel of strains with varying levels of CwpV expression was assessed by SDS-PAGE and Coomassie staining, with 630 FliC running at ∼33 kDa. Lanes 1: WT, 2: ΔcwpV, 3: pOE Nter, 4–8: pOE I-V, 9: ΔrecVON, 10: ΔrecVOFF. (B) To assess swimming motility of C. difficile strains, BHI containing 0.175% agar was inoculated to a defined depth with C. difficile from overnight liquid cultures. Tubes were incubated overnight then photographed to document motility. Tubes are numbered in the same way as the lanes in A. All cultures appeared to be equally motile. (TIF) [file ppat.1002024.s002.tif]
