# Supplementary material for: The Clostridium difficile Cell Wall Protein CwpV is Antigenically Variable between Strains, but Exhibits Conserved Aggregation-Promoting Function
Source: PLoS Pathog. 2011 Apr 21;7(4):e1002024. doi: 10.1371/journal.ppat.1002024 (PMC3080850; doi:10.1371/journal.ppat.1002024)
Supplement: Table S1 — Primers used in this study. (DOC) [file ppat.1002024.s003.doc]

Table S1. Primers used in this study

| **Name** | **Sequence (5’ to 3’)** | **Characteristic or use** |
| --- | --- | --- |
| **NF346** | CCGCTCGAGCTACACATATTTTTTAACTTCAAATGTTG | Amplify *cwpV* type I repeat unit (Fig 3Aii); |
| **NF373** | TGATAGCCCAGAAGATATTTTTCC | Amplify *cwpV* type I repeat region (Fig 3Avii) |
| **NF374** | GCCGGATCCGCAGCGCCAGCAGTA | Amplify *cwpV* type I repeat unit PCR (Fig3Aii) |
| **NF401** | TGCAGCAATCTTCTTTGGTATTTTC | Inverse PCR for sequencing *cwpV* genes from unsequenced strains |
| **NF654** | TGGTGAAAAAGGACTTGCAG | Amplify within *cwpV* cell wall binding region (Fig 3Ai), and inverse PCR for sequencing *cwpV* genes from unsequenced strains |
| **NF655** | CATCACCATTAGGCATGTTG | Amplify within *cwpV* cell wall binding region; (Fig 3Ai) |
| **NF658** | ACAAATCTGGTGTAACACAAAAAGA | Amplify 5’ end of cwpV in R20352 for sequencing |
| **NF659** | GAAGGATAAGATTAAGATTAAGGTTGC | Amplify 5’ end of cwpV in R20352 for sequencing |
| **NF701** | GCCGGATCCGGATCAAAAGCAACGTATAAAAC | Amplify *cwpV* type II repeat unit (Fig 3Aiii) |
| **NF702** | CCGCTCGAGTACAGCATAGTTTCCTTCTGCTGTTC | Amplify *cwpV* type II repeat unit (Fig 3Aiii) |
| **NF703** | AATGCAATTGTAGTAGGTACTTTCC | Amplify *cwpV* type II repeat region (Fig 3Avii) |
| **NF795** | CAATAAGTAGTGTTAAGGGAGATAA | Amplify *cwpV* repeat region (forward primer for all types) (Fig 3Avii) |
| **NF796** | AAAAATATTTACTGCTATTCTACCC | Amplify *cwpV* type III repeat unit (Fig 3Aiv) |
| **NF797** | TGTTCTGGCTGGTACTGTTAC | Amplify *cwpV* type III repeat unit (Fig 3Aiv) |
| **NF798** | GCTATAGATACAGAACAATTGG | Amplify *cwpV* type III repeat region (Fig 3Avii) |
| **NF799** | CATTTGTAAGATTTGTTTTTAATGT | Amplify *cwpV* type IV repeat unit (Fig 3Av) |
| **NF800** | TAAATGCTGCTGCTTTTGTG | Amplify *cwpV* type IV repeat unit (Fig 3Av) |
| **NF801** | AAGTAGATGCAAAAGATGTAGTG | Amplify *cwpV* type IV repeat region (Fig 3Avii) |
| **NF823** | GCATTTTTCCATCCATCTTG | *cwpV* DNA switch orientation PCR |
| **NF825** | TTTAAGGTAAGTTTGATTTTTATGTTAATGAAT | *cwpV* DNA switch orientation PCR |
| **NF826** | ATTCATTAACATAAAAATCAAACTTACCTTAAA | *cwpV* DNA switch orientation PCR |
| **NF878** | ACCAATTGTTGATATAGCACTTA | Amplify *cwpV* type V repeat unit (Figure 3Avi) |
| **NF879** | TATGTGGCACCAGTAGTGAACG | Amplify *cwpV* type V repeat unit (Figure 3Avi) |
| **NF880** | TTCTTCTACTTTATATGTCTTTCCATTTAC | Amplify *cwpV* type V repeat region (Figure 3Avii) |
| **NF1021** | GATATACCATGGGCTCATCAAAAGTAGATAAAGTAGTTTTG | Amplify R20352 type II repeats for pET28a cloning |
| **NF1022** | GTGGTGCTCGAGTACTACTTTTCCTGCTTCAAATGTCTC | Amplify R20352 type II repeats for pET28a cloning |
| **NF1023** | GATATACCATGGGCGTGGCAAAAATAACAAGCGTTCCTGC | Amplify CDKK167 type III repeats for pET28a cloning |
| **NF1024** | GTGGTGCTCGAGTATAGCTGTTCTGGCTGG | Amplify CDKK167 type III repeats for pET28a cloning |
| **NF1025** | GATATACCATGGGCACACCAACAGTAGACCCAGTTGAAGG | Amplify M9 type IV repeat for pET28a cloning |
| **NF1026** | GTGGTGCTCGAGTGTTGGTGTTTTATCTAATACTATTG | Amplify M9 type IV repeat for pET28a cloning |
| **NF1027** | GATATACCATGGGCCCAGCCAAATCAACAAAAGTAATAATAGG | Amplify AY1 type IV repeat for pET28a cloning |
| **NF1028** | GTGGTGCTCGAGTCCTGCTGTTGCTCCTGATAC | Amplify AY1 type IV repeat for pET28a cloning |
| **NF1096** | CCGGAATTCAAATGGACAAGGGGGAAGAAAATAAAATG | Amplification of *cwpV* genes for cloning into *C. difficile* expression vector |
| **NF1215** | CTTAATCACATATATCACACTTGTATAAGTTTACTGG | Analysis of *recV* ClosTron insertion site |
| **NF1296** | CACAATTTGAAAAATAAATAATGAAATATGAATAAAAATTAG | To add strep tag to *cwpV* in pCBR044 |
| **NF1297** | GATGACTCCACTCGAGTTTTAATATATTAAATAAACTTGATAGC | To add strep tag to *cwpV* in pCBR044 |
| **NF1298** | CGCCTCGAGTTTCATAAATCCTAGTATTCTTGATAACATTAATGC | Amplification of R20352 *cwpV* gene for cloning into *C. difficile* expression vector |
| **NF1299** | CGCCTCGAGTTTATTAAAATTAAAAATATTTACTGCTATTCTACCC | Amplification of CDKK167 *cwpV* gene for cloning into *C. difficile* expression vector |
| **NF1300** | CGCCTCGAGATTAATTACAGATTTTGCTACATTTGTAAGATTTG | Amplification of M9 *cwpV* gene for cloning into *C. difficile* expression vector |
| **NF1301** | CGCCTCGAGTTTAACCAGATTAGTAAACTTAAGCATTAGTC | Amplification of AY1 *cwpV* gene for cloning into *C. difficile* expression vector |
| **NF1356** | CATTAATAGCTTGCTGAGTTACCCTTATATATCGC | Analysis of *recV* ClosTron insertion site |
| **NF1357** | AGAGCTCAATCAAACTAAGAGGAGTGGTTGAAAATGGC | Amplification of *recV* for cloning |
| **NF1358** | TGGATCCTTAACCAATAAAGAAATTTTCACTAGCTTC | Amplification of *recV* for cloning |
| **NF1411** | GATTTATAAGGGTAACTCAGCAAGC | Y176F site-directed mutagenesis of *recV* |
| **NF1412** | GCTGTGTAGTGGCTACAGAGC | Y176F site-directed mutagenesis of *recV* |
