# Supplementary material for: The Clostridium difficile Cell Wall Protein CwpV is Antigenically Variable between Strains, but Exhibits Conserved Aggregation-Promoting Function
Source: PLoS Pathog. 2011 Apr 21;7(4):e1002024. doi: 10.1371/journal.ppat.1002024 (PMC3080850; doi:10.1371/journal.ppat.1002024)
Supplement: Table S2 — Plasmids used in this study. (DOC) [file ppat.1002024.s004.doc]

Table S2. Plasmids used in this study

| **Plasmid number** | **Plasmid name** | **Source** | **Description** |
| --- | --- | --- | --- |
| pMTL960 |  | [2] | *E. coli – C. difficile* shuttle vector |
| pLRP028 | pMTL007C-E2 recV-424s | This study | *recV*-targeted ClosTron plasmid |
| pCBR044 |  | [3] | pMTL960 containing the full-length *cwpV* gene from 630 |
| pCBR066 | pOENter | This study | pMTL960 containing the truncated *cwpV* gene from 630 |
| pCBR069 |  | This study | pET28a containing type II repeats from R20352 |
| pCBR070 |  | This study | pET28a containing type III repeats from CDKK167 |
| pCBR071 |  | This study | pET28a containing type IV repeat from M9 |
| pCBR072 |  | This study | pET28a containing type V repeat from AY1 |
| pCBR080 | pOEI | This study | pMTL960 containing the full-length *cwpV* gene from 630 with C-terminal strep-tag |
| pCBR105 | pOEII | This study | pMTL960 containing the full-length *cwpV* gene from R20352 with C-terminal strep-tag |
| pCBR106 | pOEIII | This study | pMTL960 containing the full-length *cwpV* gene from CDKK167 with C-terminal strep-tag |
| pCBR107 | pOEIV | This study | pMTL960 containing the full-length *cwpV* gene from M9 with C-terminal strep-tag |
| pCBR109 | pOEV | This study | pMTL960 containing the full-length *cwpV* gene from AY1 with C-terminal strep-tag |
| pCBR113 | pRecV+ | This study | pMTL960 containing *recV* from 630 |
| pCBR115 | pRecV_Y176F_+ | This study | pMTL960 containing *recV*_Y176F_ |
