# Supplementary material for: The Clostridium difficile Cell Wall Protein CwpV is Antigenically Variable between Strains, but Exhibits Conserved Aggregation-Promoting Function
Source: PLoS Pathog. 2011 Apr 21;7(4):e1002024. doi: 10.1371/journal.ppat.1002024 (PMC3080850; doi:10.1371/journal.ppat.1002024)
Supplement: Table S3 — Genetically modified C. difficile strains used in this study. (DOC) [file ppat.1002024.s005.doc]

**Table S3. Genetically modified *C. difficile*** strains used in this study

| **Strain number** | **Strain name** | **Source** | **Description** |
| --- | --- | --- | --- |
| NF2015 | 630Δ*erm* (WT) | [4] | erythromycin-sensitive derivative of *Clostridium difficile* strain 630 |
| NF2147 | WT(pMTL960) | This study | 630Δ*erm* containing pMTL960 |
| NF2185 | Δ*recV* OFF | This study | 630Δ*erm*Δ*recV* with *cwpV* DNA switch in OFF orientation |
| NF2195 | Δ*recV* ON | This study | 630Δ*erm*Δ*recV* with *cwpV* DNA switch in ON orientation |
| NF2183 | Δ*recV*(pRecV+) | This study | 630Δ*erm*Δ*recV* containing pRecV+ |
| NF2194 | Δ*recV*(pRecV_Y176F_+) | This study | 630Δ*erm*Δ*recV* containing pRecV_Y176F_+ |
| NF2078 | Δ*cwpV* | [3] | 630Δ*erm*Δ*cwpV* |
| NF2149 | Δ*cwpV*(pMTL960) | This study | 630Δ*erm*Δ*cwpV* containing pMTL960 |
| NF2167 | Δ*cwpV*(pOEI) | This study | 630Δ*erm*Δ*cwpV* containing pOE I |
| NF2176 | Δ*cwpV*(pOEII) | This study | 630Δ*erm*Δ*cwpV* containing pOE II |
| NF2177 | Δ*cwpV*(pOEIII) | This study | 630Δ*erm*Δ*cwpV* containing pOEIII |
| NF2178 | Δ*cwpV*(pOEIV) | This study | 630Δ*erm*Δ*cwpV* containing pOEIV |
| NF2179 | Δ*cwpV*(pOEV) | This study | 630Δ*erm*Δ*cwpV* containing pOEV |
| NF2151 | Δ*cwpV*(pOENter) | This study | 630Δ*erm*Δ*cwpV* containing pOENter |
