# Supplementary material for: The Clostridium difficile Cell Wall Protein CwpV is Antigenically Variable between Strains, but Exhibits Conserved Aggregation-Promoting Function
Source: PLoS Pathog. 2011 Apr 21;7(4):e1002024. doi: 10.1371/journal.ppat.1002024 (PMC3080850; doi:10.1371/journal.ppat.1002024)
Supplement: Text S1 — References for Figure S2 and Tables S2 and S3. (RTF) [file ppat.1002024.s006.rtf]

Text S1
References for Figure S2, Tables S2 and S3.
1. Twine SM, Reid CW, Aubry A, McMullin DR, Fulton KM, et al. (2009) Motility and flagellar glycosylation in Clostridium difficile. J Bacteriol 191: 7050-7062.
2. Purdy D, O'Keeffe TA, Elmore M, Herbert M, McLeod A, et al. (2002) Conjugative transfer of clostridial shuttle vectors from Escherichia coli to Clostridium difficile through circumvention of the restriction barrier. Mol Microbiol 46: 439-452.
3. Emerson JE, Stabler RA, Wren BW, Fairweather NF (2008) Microarray analysis of the transcriptional responses of Clostridium difficile to environmental and antibiotic stress. J Med Microbiol 57: 757-764.
4. Hussain HA, Roberts AP, Mullany P (2005) Generation of an erythromycin-sensitive derivative of Clostridium difficile strain 630 (630∆erm) and demonstration that the conjugative transposon Tn916E enters the genome of this strain at multiple sites. J Med Microbiol 54: 137-141.
